# Epidemiology of Pediatric Head Trauma in Guilan

**DOI:** 10.5812/atr.5289

**Published:** 2012-06-01

**Authors:** Shahrokh Yousefzadeh Chabok, Sara Ramezani, Leila Kouchakinejad, Zahra Saneei

**Affiliations:** 1 Guilan Road Trauma Research Center, Guilan University of Medical Sciences, Rasht, IR Iran

**Keywords:** Epidemiology, Head Injuries, Pediatric Trauma, Traffic Accidents

## Abstract

**Background:**

Head injury (HI) is preventable and knowledge of the epidemiology of children's HI is essential for developing preventive strategies.

**Objectives:**

The aim of this study was to survey pediatric HI patients admitted to emergency wards at Poursina Hospital in Rasht, Iran, from 2009 to 2010, and to identify the cause of HI in these children.

**Patients and Methods:**

In this retrospective study, all HI patients under the age of 18 who were admitted to emergency wards between March 2009 and March 2010 were enrolled in the study. Demographic, etiologic, and injury data were collected and a descriptive analysis was performed.

**Results:**

A total of 668 patients were included in this study. The mean age was 10.4 ± 5.3 years. The most frequent cause of HI was traffic accidents. The mean Glasgow Coma Scale (GCS) score was 14.5 ± 1.6. The ratio of boys to girls was approximately 3 to 1. The ratio of boys to girls increased with increasing age (*P *< 0.01). Moreover, an association was found between age at injury and etiology of HI as well as a significant association between age at injury and the place of event (*P *< 0.01).

**Conclusions:**

The incidence of childhood HI due to traffic accidents is high (81% of pediatric trauma cases). Thus, motorcyclist education and improvement in traffic engineering for pedestrians and bicyclists should be included in prevention programs.

## 1. Background

Head injuries (HIs) are a very common problem in the pediatric population. They are one of the primary causes of injury mortality and morbidity in childhood.

 Trauma is a leading cause of death in children older than 1 year in the United States, with HI representing 80% or more of those injuries. In approximately 5% of HI cases, the patient dies at the site of the accident. HI has a high emotional, psychosocial, and economic impact because these patients often have comparatively long hospital stays, and 5%-10% of them require long-term care after discharge (1).

Each year, an estimated 475,000 traumatic brain injuries (TBIs) occur among children aged 0-14 years in the US. With nearly half a million children affected each year, TBI is a serious public health problem. Rates are highest among children aged 0-4 years. The mechanisms of HI in children vary depending on age. The younger the child, the higher the risk of HI, because of their large heads, weak neck musculature, and relatively thin calvarium (2, 3).

Children are especially vulnerable to traffic injuries, particularly in developing countries. In the low-to-middle income countries of the Americas, road traffic injuries (RTIs) are the leading cause of death and morbidity among children aged 5-14 years, and a major cause of death among children aged 0-4 years (4). Injuries from falling, drowning, and burning are the second, third, and fourth most common causes of death in children, respectively (5). According to the World Health Organization, falls ranked as the world's fifth most common cause of death in children aged 5-14 years in the year 2000, and falling is the most frequent type of accidental injury. It is interesting to note that these types of injuries mainly occur in the home, since it is the place where children spend most of their time (6, 7).

## 2. Objectives

The aim of this study was to investigate the individual and environmental determinants of HI across pediatric age groups in Guilan province.

## 3. Patients and Methods

A retrospective study was carried out on patients under the age of 18 who were admitted for HI to the emergency wards at Poursina Hospital in Guilan province, Iran, from March 2009 to March 2010. Clinical findings, Glasgow Coma Scale (GCS) and radiographic findings, length of stay, demographic data (age, gender, and education), anatomic region, and etiology of injury were recorded. An environmental risk factor study evaluated vehicle and pedestrian movement and the place of injury. Those cases in which the intracranial lesion was of medical origin or secondary to another disease process were excluded from further statistical analysis. The pediatric patients were divided into five groups according to their age (infants: 0-1 year, toddlers: 2-5 years, children: 6-9 years, pupils: 10-13 years, teenagers: 14-17 years) (8).

A descriptive analysis of collected data was performed using SPSS version 16.0. Regression analysis was performed to determine risk factors for head injury in children and a Chi-square analysis (χ2) was utilized to compare the sex distribution, rates of various types of injuries, and mortality rate. Results were considered significant at P < 0.05. 

## 4. Results 

From March 2009 to March 2010, a total of 668 (487 boys and 181 girls) head injury patients under the age 18 years were admitted to the emergency ward. The mean GCS was 14.52 ± 1.6. In our study, approximately 90% of the patients had no CT findings. 

**Figure 1.**
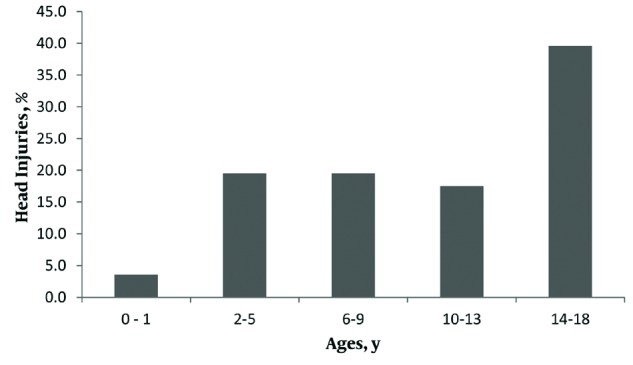
Age distribution of pediatric head Injury patients admitted to emergency wards.

The mean age of this population was 10.42 ± 5.3 years. The most common age group was 14-17 year olds. Figure 1 illustrates the age distribution of pediatric HI patients admitted to the emergency wards. Eighty-one percent of pediatric injury cases had HIs and 13% had HI without injuries to other anatomic regions. HI was more frequent in boys (72.7%) than girls (26.4%) and more frequent in rural regions (51.5%) than urban areas (47.3%). 

RTIs were the most common cause of HI (n = 441, 65%). Thirteen percent (n = 88) of all head injuries were child pedestrian RTIs. Almost 25% (n = 167; 24.7%) of HIs were caused by falling and 3.4% (n = 23) by fighting. The most common place where HI occurred was suburban roads (n = 245, 36.1%) followed by city streets (n = 222, 32.7%) and home (n = 147, 21.7%). Chi-square analysis results showed that gender proportion differed significantly by age group (χ2 = 23.02, df = 4; P < 0.01). The ratio of boys to girls increased with increasing age group. Etiology of injury differed significantly in rural and urban areas. HIs caused by agricultural machines and motorbike were more frequent in rural than urban areas but pedestrian and bicyclist HIs were more frequent in children from urban areas, (χ2 = 20.08, df = 10; P < 0.01). There was also a significant association between age at injury and the place where HI occurred (χ2 = 1.53, df = 52; P < 0.01) and age at injury and injury etiology (χ2 = 1.37, df = 40; P < 0.01). In children under the age of 1 year, falling was the most common cause of HI, whereas RTIs were the most frequent cause in older age groups. The occurrence of falls decreased with increasing age. 

Among RTIs with HI, the most frequent cause of HI was motorcycle accident (20%) for children under the age of 1 year. In the 2-5-year-old age group, child pedestrian road traffic injuries (14.5%) and motorcycle accident (13%) were the most frequent causes of RTI-associated HI. Likewise, in the 6-9-year-old age group, motorcycle accident (20%) and child pedestrian road traffic injuries (19.8%) were the most frequent causes of RTI-associated HI. In the 10-13-year-old age group, the leading cause of RTI-associated HI was bicycle accident (17%), followed by child pedestrian road traffic injuries (15.4%) and motorcycle accident (14.5%). Finally, in the 14-18-year-old age group, motorcycle accident (42%) was the most common cause of RTI-associated HI. None of the motorcyclist or bicycle riders wore a helmet. The distribution of main causes of HI by age group is shown in Table 1. 

**Table 1.** Distribution of Cause of Head Injury by Age Group

	Infants (0-1), y	Toddlers (2-5), y	Children(6-9), y	Pupils(10-13), y	Teenager(14-18), y
Traffic accident, %	25	50.6	57.4	70.9	77.7
Falling. %	70.9	41.6	34.1	1.8	10.6
Fight, %	0	0	1.6	5.1	5.7
Shotgun, %	0	0	0	1.7	0
Sport, %	0	0	0	0	1.5
Child abuse, %	0	0	0.8	0	0
Other, %	4.1	7.8	5.4	2.6	3.4
Total, %	100	100	100	100	100

## 5. Discussion

On the basis of the mean GCS score and normal CT scans, the majority of the patients included in this study sustained only mild HI. The prevalence of HI is relatively stable throughout childhood, although an increase in the incidence of head injury was identified in 2 age groups. Around the age of 15 years old, a dramatic increase in HI occurs, primarily in boys. Infants under the age of 1 year have also been identified by several studies (9- 12) as having an elevated incidence of HI, which is attributed to falls and child abuse. Fernandez et al. found that the most common etiology of head injury in children younger than 2 years old was a fall at home. In that study, child abuse accounted for 7% of HI (11). In this study, the main cause of HI in infants under the age of 1 year was also falling (Table 1). Child abuse was not reported as the cause of HI in this age group, which could be due to lack of disclosure. Stairs was the most common cause of falling in children aged 0-9 years old. Our data show that HI in younger children happens more frequently at home, whereas in older children it occurs more frequently on the road. 

Across all age groups, the ratio of boys to girls with HI was greater than one, gradually increasing with age. In our study, children aged 14-18 years had the highest incidence of HI with a boy to girl ratio of approximately 4 to 1. This may be due to gender-specific differences in driving activities and sports participation. Compared to more-developed countries, HI due to sports was rare, which may be accounted for by low sport participation in Iran.

In India, pedestrian RTIs are presumed to be the most common cause of HI and the majority of those injured are children (13-15). This contrasts with our previous study in Guilin in which the most common cause of HI was motorcycle accident (16). In the present study, RTs were the most common cause of HI (65%) and were comprised of 25% of HIs were caused by motorcycle accidents, 15% car accidents, and 13% pedestrian accidents. In a study performed in Qatar from 1993 to 2007, there was a dramatic increase in childhood mortality caused by RTIs (17). Most traffic-related HIs occur secondary to motorcycle accidents in the 14-18-year-old age group (42%) and bicycle accidents in 10-13-year-old age group (17%). In this age group (10-13 y), motorcycle accidents were 14% and in 8 cases (6.8%) the person was the driver of motorcycle. The percentage of each contributing factor differs between studies, and the distribution varies according to age, group, and gender. Rogers et al. reported that, among pediatric trauma patients in the US, only 15% of bicyclists wore helmets (18). In the population studied here, no bicyclists wore helmets. Given the finding that bicycle accidents were the most common cause of HI in children aged 10-13 years, more attention to helmet education is warranted. Our data indicated there were differences between rural and urban HIs. HIs caused by agricultural machines were frequent in rural areas, but rare in urban areas. In addition, pedestrian and bicyclist HIs were more frequent in children from urban areas, whereas HIs caused by motorbike accident were more frequent in children from rural areas. Pediatric HI remains a major threat to the health and well-being of children, and has an enormous impact on society and hospital workload. There is a need for more investigation of environmental factors, cultural factors, and determinants of head injury in children, particularly with regard to traffic-related HIs. Given that pedestrian RTIs and motorcycle accidents were common, education on the use of seat belts for motorists, education on the use of helmets for motorcyclists and bicyclists, and traffic engineering for the benefit of pedestrians, could be useful in reducing HI incidence and severity.

## References

[1] Cakmakci H (2009). Essentials of trauma: head and spine.. Pediatr Radiol..

[2] Langlois JA, Rutland-Brown W, Thomas KE (2005). The incidence of traumatic brain injury among children in the United States: differences by race.. J Head Trauma Rehabil..

[3] Murgio A (2003). Epidemiology of traumatic brain injury in children.. Revista Espanola de neuropsicologia..

[4] Donroe J, Tincopa M, Gilman RH, Brugge D, Moore DA (2008). Pedestrian road traffic injuries in urban Peruvian children and adolescents: case control analyses of personal and environmental risk factors.. PLoS One..

[5] Peden M, Oyegbite K, Ozanne-Smith J, Hyder AA, Branche C, Rahman AKMF World Report on child injury prevention. Geneva. 2008. http://whqlibdoc.who.int/publications/2008/9789241563574_eng.pdf.

[6] Britton JW (2005). Kids can't fly: preventing fall injuries in children.. WMJ..

[7] Peden MM, McGee K, Krug E, Injuries WHO, Dept VP (2002). Injury: a leading cause of the global burden of disease, 2000..

[8] Nau C, Jakob H, Mark L, Schneidmüller D, Marzi I, Laurer H (2010). Epidemiology and Management of Injuries to the Spinal Cord and Column in Pediatric Multiple-Trauma Patients.. Eur J Trauma Emerg Surg..

[9] Allard RH, van Merkesteyn JP, Baart JA (2009). Child abuse.. Ned Tijdschr Tandheelkd..

[10] Case ME (2008). Accidental traumatic head injury in infants and young children.. Brain Pathol..

[11] Fernandez ML, Mejias L, Ortiz N, Garcia-Fragoso L (2010). Minor head injury in children younger than two years of age: description, prevalence and management in the emergency room of the pediatric university hospital.. Bol Asoc Med P R..

[12] Oehmichen M, Meissner C, Saternus KS (2005). Fall or shaken: traumatic brain injury in children caused by falls or abuse at home - a review on biomechanics and diagnosis.. Neuropediatrics..

[13] Patel M, Kartha G, Mehta HK (2010). An analysis of pedestrian accidents in Sabarkantha district. National J Community Med..

[14] Singh H, Dhattarwal SK, Mittal S, Aggarwal A, Sharma G, Chawla R (2007). A review of Pedestrian Traffic Fatalities.. J Indian Acad Forensic Med.

[15] Tabish A, Lone NA, Afzal WM, Salam A (2006). The incidence and severity of injury in children hospitalised for traumatic brain injury in Kashmir.. Injury..

[16] Yousef zadeh Chabok S, Safayi M, Hemati H, Mohammadi H, Ahmadi dafchahi M, L K (2008). Epidemiology of Head Injury in Patients who were Reffered to Poorsina Hospital.. J Guilan Univ Medi Sci.

[17] Bener A, Hussain SJ, Ghaffar A, Abou-Taleb H, El-Sayed HF (2011). Trends in childhood trauma mortality in the fast economically developing State of Qatar.. World J Pediatr..

[18] Rogers SC, Campbell BT, Saleheen H, Borrup K, Lapidus G (2010). Using trauma registry data to guide injury prevention program activities.. J Trauma..

